# Cholestasis and disseminated histoplasmosis in a psoriatic patient on infliximab: case report and review of literature

**DOI:** 10.1186/s12876-020-01290-3

**Published:** 2020-05-08

**Authors:** Steven Park, Janice Cheong, Kaitlin Kyi, Jose Aranez, Sohaib Abu-Farsakh, Christa Whitney-Miller, Bandar Al-Judaibi, Marie Laryea

**Affiliations:** 1 Department of Internal Medicine, University of Rochester Medical Center, 601 Elmwood Avenue, P.O. Box MED, Rochester, NY 14642 USA; 2 Division of Gastroenterology and Hepatology, University of Rochester Medical Center, 601 Elmwood Avenue, Box 646, Rochester, NY 14642 USA; 3 Department of Pathology and Laboratory Medicine, University of Rochester Medical Center, 601 Elmwood Avenue, Box 626, Rochester, NY 14642 USA; 4 Department of Transplant Surgery, University of Rochester Medical Center, 601 Elmwood Avenue, Box SURG, Rochester, NY 14642 USA

**Keywords:** *Histoplasma capsulatum*, Histoplasmosis, Granulomatous hepatitis

## Abstract

**Background:**

*Histoplasma capsulatum* is the most common endemic mycosis in the United States and frequently presents as an opportunistic infection in immunocompromised hosts. Though liver involvement is common in disseminated histoplasmosis, primary gastrointestinal histoplasmosis of the liver in absence of lung involvement is rare. Similarly, cholestatic granulomatous hepatitis in liver histoplasmosis is rarely seen.

**Case presentation:**

We present a rare case of primary gastrointestinal histoplasmosis manifesting with acute granulomatous hepatitis and cholestasis in a 48-year-old female with psoriatic arthritis, receiving methotrexate and infliximab. The epidemiology, risk factors, clinical presentation, diagnosis, and treatment of histoplasmosis is discussed. Furthermore, we review the published cases of biopsy-proven disseminated histoplasmosis with cholestatic jaundice to highlight histoplasmosis involvement in the liver.

**Conclusion:**

Histoplasmosis should be considered in immunosuppressed patients with fever, chills, abdominal pain and cholestasis with progressive jaundice, particularly in subjects without evidence of biliary obstruction. Future studies are needed to accurately assess the risk of this fungal infection, specifically in patients on immunomodulatory therapy for autoimmune disease.

## Background

Histoplasmosis occurs in many areas around the world but is endemic to the Mississippi and Ohio River valleys in the United States [1]. It is caused by *Histoplasma capsulatum*, a dimorphic fungus found in barns, old houses, and soil rich in bird and bat droppings. Direct inhalation of spores is the primary mode of infection. Symptomatic infection with *H. Capsulatum* is most likely in patients who are immunosuppressed and unable to develop an effective cell-mediated immunity against the organism [2]. Primarily a pulmonary disease, histoplasmosis presents either acutely or chronically with a range from organ-specific disease to disseminated disease [3]. Gastrointestinal histoplasmosis is rare and often presents as a diagnostic dilemma [4]. Though liver involvement is common in disseminated histoplasmosis, liver histoplasmosis as an initial sign of histoplasmosis without lung involvement is rare. In particular, cholestasis due to *H. capsulatum* in the setting of primary liver manifestation has been rarely observed. We offer a case in an immunosuppressed patient who presented with acute cholestatic granulomatous hepatitis and was found to have disseminated histoplasmosis.

## Case presentation

A 48-year-old female with psoriatic arthritis on methotrexate and infliximab was transferred to our hospital for evaluation of persistent fever, right-upper-quadrant (RUQ) pain and elevated liver enzymes. Two days prior to presentation, the patient underwent an elective laparoscopic cholecystectomy for biliary colic. However, her RUQ pain persisted and she became febrile. The patient denied recent travel or significant smoking or alcohol use. Family history was notable for psoriasis, autoimmune hepatitis and non-alcoholic fatty liver disease.

Laboratory data revealed ALT 218 U/L [ref. range 0–35 U/L], AST 181 U/L [ref. range 0–35 U/L], ALP 1138 U/L [ref. range 35–105 U/L], and LDH 406 U/L [ref. range 118–225 U/L]. On admission to our hospital, additional laboratory investigation was notable for elevated white blood cell count of 13.5 K/μL (ref. range 4–10 K/μL), lymphocyte count 8.5 K/μL (ref. range 1.2–3.7 K/μL), GGT 885 U/L (ref. range 5–36), total bilirubin 2.5 mg/dL (ref. range 0–1.2), and ferritin 1229 ng/ml (ref. range 10–120 ng/ml). An abdominal ultrasound showed non-specific post-cholecystectomy changes; a hepatobiliary iminodiacetic acid (HIDA) scan was negative for biliary leak or obstruction; computed tomography (CT) of abdomen and pelvis with contrast was without focal liver lesions or fluid collections; and an magnetic resonance cholangiopancreatography (MRCP) was without intrahepatic biliary ductal dilatation. The patient was then transferred to our institution for further evaluation.

Serologic testing for hepatitis viruses A, B and C, Epstein-Barr virus, cytomegalovirus, herpes simplex virus and human immunodeficiency virus were negative. A high titer of anti-nuclear antibodies (ANA) 1:640 was detected while Ig immunoglobulins and rheumatoid factor were within normal range. F-actin IgG and anti-Histone antibodies were weakly positive at 32 [ref. range 0–19 units] and 2.6 [ref. range 0–0.9], respectively. Anti-neutrophil cytoplasmic, anti-RNP, anti-Smith, anti-SSA/SSB and anti-dsDNA autoantibodies were negative.

**Fig. 1:**
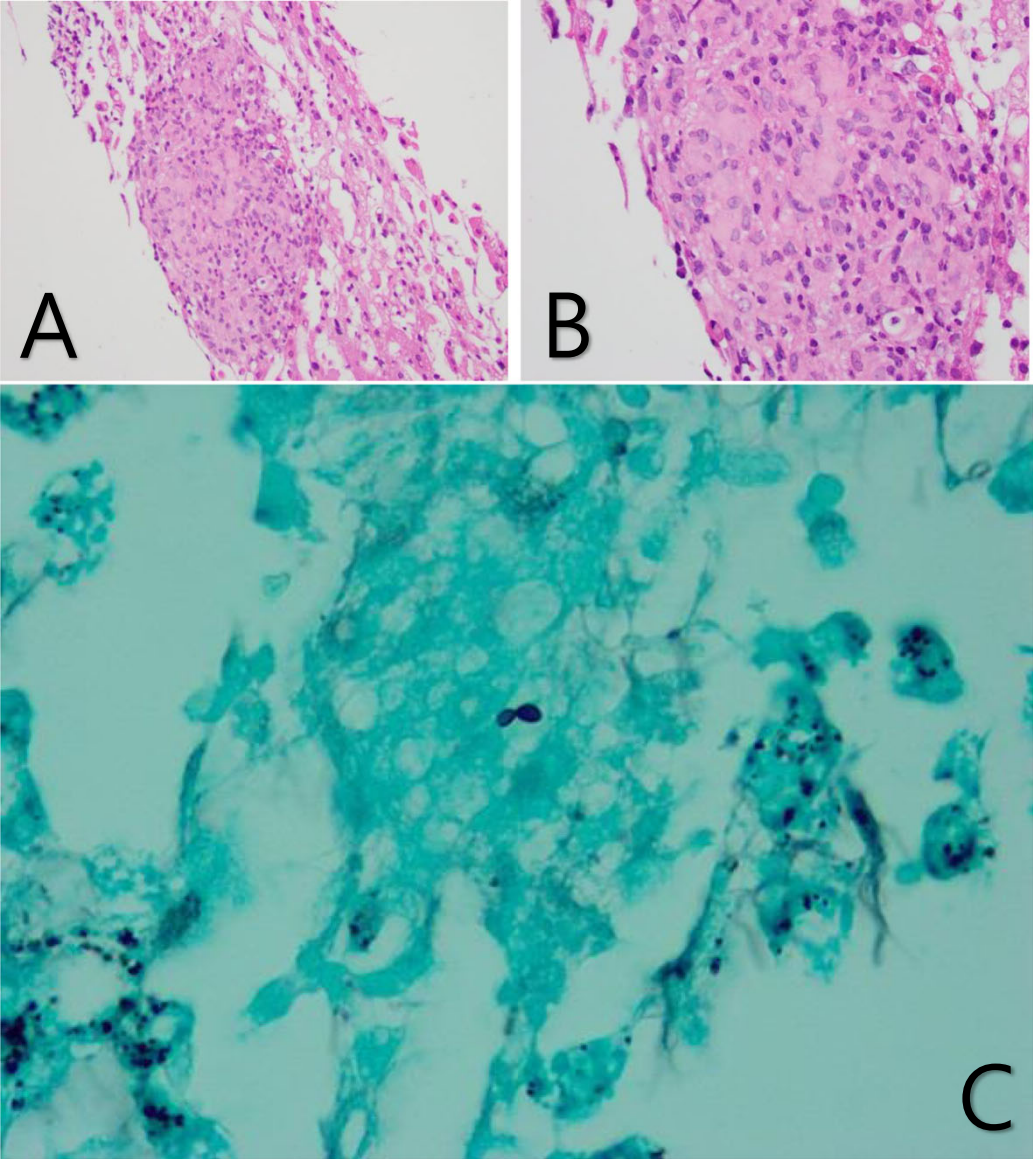
Histology of *Histoplasma var. Capsulatum.***a** H&E stain on 20x magnification demonstrating non-necrotizing granuloma with sinusoidal congestion. **b** H&E stain on 40x magnification demonstrating the non-necrotizing granuloma. **c** GMS stain demonstrating budding yeast

The direct bilirubin reached 6.0 mg/dL (ref. range 0–0.3 mg/dL) on day 6 of admission despite normal indirect bilirubin of 0.5 mg/dL (ref. range 0.1–1.0 mg/dL). A liver biopsy was obtained on Day 7. This found numerous non-necrotizing granulomas with sinusoidal congestion, mild predominantly microvesicular steatosis (~ 20%) without significant ductitis or ductular reaction, no fibrosis on H&E stain, rare small budding yeast on GMS stain, negative acid-fast stain, negative PAS-D stain, negative iron stain, and minimal pericellular and periportal fibrosis on trichrome stain (Fig. 1). The patient was then started on antifungal treatment with Amphotericin B.

Serum and urine *Histoplasma capsulatum* antigen came back as positive on Day 15 and the patient’s direct bilirubin peaked at 11.8 mg/dL on day 16 of admission. After 2 weeks of IV amphotericin B, patient was transitioned to PO itraconazole 200 mg BID on day 23 of admission for a planned 1-year duration. During the remaining course of her hospitalization, she developed significant left-sided pleural effusions requiring two thoracenteses. On both occasions, pleural fluid was exudative. Medical cytology was negative for malignant cells, and culture data was negative. She then developed a significant cardiac tamponade requiring an urgent pericardiocentesis, once again without malignant cells or positive cultures. The patient’s liver function tests normalized after 68 days of treatment and she was discharged with a long-term course of itraconazole.

## Discussion and conclusions

Histoplasmosis is the most common endemic mycosis in the United States, in the environment in areas surrounding the Ohio and Mississippi River valleys. Between 60 and 90% of people who live in this area have been exposed to the fungus at some point during their lifetime (https://www.cdc.gov/fungal/diseases/histoplasmosis/statistics.html). The incidence of histoplasmosis in adults aged 65 years and older in the U.S. is 3.4 cases per 100,000 population and up to 6.1 cases per 100,000 population [5]. Worldwide, histoplasmosis is most commonly an opportunistic infection in the context of HIV infection or immunosuppression [6].

Gastrointestinal (GI) tract involvement has been reported in histoplasmosis but is usually asymptomatic or presents with vague abdominal pain in the majority of patients [7]. GI manifestations occur only in 3–12% of patients with colon being the most commonly involved site followed by the small bowel [8–10]. Though *H. capsulatum* is an uncommon cause of granulomatous hepatitis, liver is often involved in the course of progressive disseminated disease. However, hepatic manifestation being the primary presenting symptom is uncommon. Patients with primary liver involvement present with nonspecific symptoms such as fever, nausea, vomiting, fatigue, weight loss, and elevation of liver function tests, as seen in our patient. In patients with underlying acquired immune deficiency syndrome (AIDS), anorectal diseases, and GI bleeding associated with superficial or deep ulcers with and without perforation are more common compared to immunocompetent individuals [4]. Immunosuppression also increases the risk of colonic obstruction due to large inflammatory masses that may mimic malignancy [4].

The diagnosis of histoplasmosis involves a multifaceted approach involving clinical and laboratory evidence of disease [11]. The gold standard for laboratory diagnosis of H. capsulatum is identifying the yeast with tissue stains or mold on cultured specimen. In clinical practice, the non-invasive and widely accessible antigen detection is preferred for initial testing. The MiraVista enzyme immunoassay (EIA) Histoplasma antigen test boasts a sensitivity of 91.8% in patients with disseminated histoplasmosis, 87.5% in patients with chronic pulmonary histoplasmosis and 83% in patients with acute pulmonary disease [12]. The combination of urine and serum antigen testing improves the efficacy of testing and the latter is particularly useful for monitoring the treatment response [13]. Without a high clinical suspicion, however, a liver biopsy can provide a histopathological evidence in support of diagnosis of histoplasmosis. On histology, *H.capsulatum var. capsulatum* appears as ovoid yeast cells measuring 2 to 4 μm with narrow based budding. Hematoxylin and eosin (H&E) stain may reveal portal tract infiltration with lymphocytes or histiocytes but is often too insensitive to detect the presence of fugal organisms. Additional staining with Gomori methenamine silver (GMS) and periodic acid-Schiff (PAS) best detects *H.capsulatum* by highlighting the yeast cell wall. As the presence of yeast cells consistent with *H.capsulatum* may not necessarily indicate active infection, culture data should be interpreted in the appropriate clinical context. Fungal culture can take up to 8 weeks for colony identification and its sensitivity for progressive disseminated histoplasmosis and chronic pulmonary disease is 74.2 and 66.7%, respectively [11]. Furthermore, examination of tissue aspirates and fluids (e.g., bronchoalveolar lavage fluid) with GMS or PAS staining can reveal narrow-based budding yeasts within macrophages. In our case, liver biopsy was performed prior to the antigen detection. Positive findings on biopsy were numerous randomly positioned non-necrotizing granuloma on H&E stain and rare small budding yeast on GMS stain. These findings led to urine and serum antigen detection which both resulted positively.

Since disseminated histoplasmosis is fatal without treatment, prompt initiation of antifungal therapy is essential. Amphotericin B and itraconazole are the two agents most commonly used to treat for disseminated histoplasmosis [14]. However, mild pulmonary disease can be treated with itraconazole. Alternative treatment options include fluconazole, which is not as active as itraconazole against *H.capsulatum*, voriconazole and posaconazole. As mentioned previously, serum and urine antigen levels are useful for monitoring treatment with most decline appreciated in the first 2 weeks of successful therapy and continued for 12 weeks.

Our patient case illustrates an uncommon presentation of histoplasmosis that began as acute granulomatous hepatitis associated with cholestatic jaundice and progressed to disseminated histoplasmosis. Despite starting the appropriate treatment with IV Amphotericin and subsequently switched to oral Itraconazole, our patient developed worsening cholestasis as well as systemic symptoms such as pleural effusions and cardiac tamponade. After multiple thoracenteses and an urgent pericardiocentesis, patient recovered with full normalization of liver function tests by the 68th day of treatment.

With an extensive laboratory and imaging work-up, making the diagnosis of histoplasmosis is often challenging. In our case, the liver biopsy showed the pathognomonic findings of histoplasmosis including granulomatous hepatitis and ovoid yeast elements.

Cholestasis has received little attention in case reports of disseminated histoplasmosis. This is likely because not all cases of hepatic histoplasmosis manifest with significant cholestasis [15]. Thus far, only a few case reports of liver-biopsy proven histoplasmosis have reported associated cholestasis [16–20]. As shown in Table 1, the typical clinical presentations are fever and jaundice in the setting of immunosuppression. The range of laboratory values associated with disseminated histoplasmosis is highly variable. Transaminase levels are usually high in the 100 s, but transaminitis may be absent upon presentation. Elevated alkaline phosphatase level is often one of the most prominent laboratory findings with levels as high as > 2100 U/L. However, an ALP level into the 300 s can also be associated with histoplasmosis. In addition, high total bilirubin is usually associated with a concomitant rise in direct bilirubin. GGT (γ-glutamyl transpeptidase), when obtained, is significantly elevated as demonstrated by the case reported by Gill et al. and our patient [20]. A liver biopsy is often pursued in conjunction with serum and urine antigen studies in order to establish the diagnosis. The PAS and GMS stain on histology effectively detects *H.capsulatum* by highlighting the yeast cell wall. Furthermore, Kothadia and colleagues described a case of granulomatous hepatitis in an HIV patient who initially presented with a total bilirubin of 10.2 mg/dL and succumbed to severe infection with multiorgan system failure within 3 weeks of admission [19]. The severity of cholestasis may serve as a prognostic marker in disseminated histoplasmosis.

**Table 1:** Summary of Case Reports of Cholestasis in Disseminated Histoplasmosis

Source	Brief Description of Patient and Clinical Presentation	Laboratory Values	Diagnostic Investigations	Liver Biopsy Findings	Tx and Complications
*Wee* et al. (2009) [16]	59 year old male with recent travel to Indonesian farmland presented with 1 month of fever, icterus and tea colored urine	AST&ALT normal ALP 528 U/L T.Bili 15.2 mg/dL D.Bili 11.3 mg/dL	+ Serum Ab + Urine Antigen (8.45 EIA) + BCx *H.Capsulatum*	GMS: intracellular budding yeast 2–3 μm in diameter [Verified with bone marrow aspirate]	IV Amphotericin B (dose and duration not presented) without complications
*van Welzen* et al. (2013) [18]	74 year old female with history of necrotizing scleritis on prednisone, methotrexate and adalimumab presented with shortness of breath	AST 129 U/L ALT 111 U/L ALP > 2100 U/L T.Bili 3.7 mg/dL D.Bili 2.2 mg/dL	+ PCR and culture with liver tissue specimen, colonic tissue specimen, and bronchial fluid + BCx *H.Capsulatum* - Serum Ab	H&E: portal infiltrates composed of lymphocytes, histiocytes and multinuclear histiocytic cells PAS: multinuclear histiocytic cells containing fungal organisms GMS: multinuclear histiocytic cells containing fungal organisms	IV Amphotericin B for 2 weeks, then Itraconazole 200 mg BID for 1 year. The patient’s hospitalization was complicated by hematochezia.
*Rihana* et al. (2014) [17]	66 year old female with history of rheumatoid arthritis on methotrexate on infliximab with recent travel to Kansas presented with 3 weeks of fever, chills, tachycardia, and painless jaundice	AST 173 U/L ALT 252 U/L ALP 375 U/L T.Bili 4.2 mg/dL	+ Serum Antigen > 19 ng/mL + Cx on Bronchilolar Lavage	H&E: fungal organisms within areas of granulomatous inflammation GMS: round to ovoid 2–4 μm narrow based budding yeast Acid Fast: negative Immunohistochemical: negative	IV Amphotericin B was started, then due to acute kidney injury, was changed to Itraconazole. This was stopped and changed to Voriconazole due to GI bleed
*Gill* et al. (2017) [20]	61 year old female with history of rheumatoid arthritis presented with fever, chills, abdominal pain and jaundice while on hydroxychloroquine	AST 449 U/L ALT 745 U/L ALP 1045 U/L T.Bili 11.6 mg/dL D.Bili 2.4 mg/dL GGT 620 U/L	+ BCx *H.Capsulatum* + Urine Antigen	*Liver biopsy* not performed	IV Amphotericin B + Voriconazole were started. The patient was discharged on Itraconazole
*Kothadia* et al. (2017) [19]	41 year old male with history of kidney transplant on immunosuppression presented with fever, malaise and jaundice	AST 70 U/L ALT 68 U/L ALP 1351 U/L T.Bili 10.2 mg/dL	+ Urine Antigen > 25 ng/mL + HIV	H&E: non-necrotizing granulomatous inflammation with histiocytes GMS: Round to ovoid, narrow budding yeasts	Patient passed away secondary to multiorgan failure in the setting of sepsis

Two days prior to presentation, our patient underwent an elective laparoscopic cholecystectomy for right upper quadrant pain thought to be biliary colic. In retrospect, her presentation may have been secondary to disseminated histoplasmosis. An important learning objective from our case is to have high index of clinical suspicion for histoplasmosis among immunocompromised patients who present with cholestasis in the absence of other causes by standard laboratory and imaging evaluation. The case presented by Kothadia et al. also describes a patient who underwent laparoscopic cholecystectomy for unclear etiology shortly before being diagnosed with disseminated histoplasmosis [19]. Although it is unknown how many patients with cholestatic jaundice due to disseminated histoplasmosis undergo unnecessary laparoscopic cholecystectomy, our case points to the importance to consider this etiology for the clinical picture of fever, RUQ pain and jaundice in endemic regions. In regions endemic for H. capsulatum, a combination of urine and serum antigen testing could be done for right sided abdominal pain. Given the presentation of fever, right-upper-quadrant pain, and mildly abnormal liver enzymes with hyperbilirubinemia, biliary obstruction and cholecystitis will often appropriately be in the differential. Lack of radiographic evidence of biliary disease should however prompt further investigation especially in immunocompromised patients.

Histoplasmosis should be considered in immunosuppressed patients presenting with fever, chills, abdominal pain and cholestasis with progressive jaundice, particularly in subjects without evidence of biliary obstruction. The full spectrum of hepatic manifestations of this disease is unknown but runs the gamut in the literature from mildly abnormal liver enzymes to severe icteric cholestasis with fever and pain. Future studies are needed to accurately assess the risk of this fungal infection specifically in patients on immunomodulatory therapy for autoimmune disease.

## Data Availability

All data generated or analyzed during this study are included in this published article [and its supplementary information files].

## Abbreviations

RUQ: Right upper quadrant
HIDA: Hepatobiliary iminodiacetic acid
EF: Ejection Fraction
ED: Emergency Department
ALT: Alanine transaminase
AST: Aspartate transaminase
CT: Computed Tomography
MRCP: Magnetic Resonance Cholangiopancreatography
ANA: Anti-nuclear antibody
H&E: Hematoxylin & Eosin
GMS: Gomori Methenamine Silver
RNP: Ribonucleoprotein
SSA/SSB: Sjogren’s syndrome related antigen A/B
dsDNA: Double stranded Deoxyribose nucleic acid
PAS: Periodic acid-Schiff
BID: Twice a day
HIV: Human immunodeficiency virus
AIDS: Acquired immune deficiency syndrome
EIA: Enzyme immunoassay
TNF: Tumor necrosis factor
ALP: Alkaline phosphatase
GGT: Gamma glutamyl transpeptidase
